# AqSolDB, a curated reference set of aqueous solubility and 2D descriptors for a diverse set of compounds

**DOI:** 10.1038/s41597-019-0151-1

**Published:** 2019-08-08

**Authors:** Murat Cihan Sorkun, Abhishek Khetan, Süleyman Er

**Affiliations:** 1 DIFFER - Dutch Institute for Fundamental Energy Research, De Zaale 20, 5612 AJ Eindhoven, The Netherlands; 2 Center for Computational Energy Research, DIFFER - Dutch Institute for Fundamental Energy Research, De Zaale 20, 5612 AJ Eindhoven, The Netherlands

**Keywords:** Thermodynamics, Cheminformatics

## Abstract

Water is a ubiquitous solvent in chemistry and life. It is therefore no surprise that the aqueous solubility of compounds has a key role in various domains, including but not limited to drug discovery, paint, coating, and battery materials design. Measurement and prediction of aqueous solubility is a complex and prevailing challenge in chemistry. For the latter, different data-driven prediction models have recently been developed to augment the physics-based modeling approaches. To construct accurate data-driven estimation models, it is essential that the underlying experimental calibration data used by these models is of high fidelity and quality. Existing solubility datasets show variance in the chemical space of compounds covered, measurement methods, experimental conditions, but also in the non-standard representations, size, and accessibility of data. To address this problem, we generated a new database of compounds, AqSolDB, by merging a total of nine different aqueous solubility datasets, curating the merged data, standardizing and validating the compound representation formats, marking with reliability labels, and providing 2D descriptors of compounds as a Supplementary Resource.

## Abstract (summary)

**Table:** 

Design Type(s)	chemical reaction data analysis objective • data integration objective • data validation objective
Measurement Type(s)	aqueous solubility
Technology Type(s)	digital curation
Factor Type(s)	physical state
Sample Characteristic(s)

Machine-accessible metadata file describing the reported data (ISA-Tab format)

## Background & Summary

Aqueous solubility constitutes a crucial property of chemical substances that governs behavior of phenomena in several areas like geochemistry, climate predictions, biochemistry, drug-design, agrochemical design, and protein ligand binding. It is defined as the maximum amount of a compound, i.e., the solute, that can get dissolved in a given volume of water, and depends on physical conditions such as temperature and pressure. It is of critical importance in especially pharmaceutical drug design, where poor aqueous solubility is likely to lead to precipitation of compounds from screening buffer, which may create a high risk of erroneous results, false leads, and increased costs and formulation difficulties during clinical development.

Although the aqueous solubility of a compound can be related to its other structural and physico-chemical properties such as shape, polar surface area (PSA), acid dissociation constant (pKa), lipophilicity (logD), and the number of hydrogen bond donors and acceptors, theoretical predictions are often inaccurate. In order to overcome these challenges, several data-driven models have been developed to predict the aqueous solubility of compounds last couple of decades^1–6^.

The development of reliable data-driven models, however, has been hindered by uncertainties and disagreements in the underlying data, which are obtained from many disparate sources. Unsystematic errors between different experimental methodologies potentially limit the accuracy with which the models can be trained and validated. To develop generalizable prediction models, accurate datasets are needed that are diverse and large at the same time^7^.

In this work, we assess the quality of aqueous solubility datasets under 2 categories: generalizability and fidelity. Generalizability can be interpreted in terms of the chemical diversity of the dataset, as well as its size. Machine learning models developed using datasets, which have small size and lack chemical diversity, show poor predictive capability on external test sets, as shown in the study by Wang *et al*.^8^. Another very important indicator of dataset quality is fidelity. Fidelity can be understood as accuracy of data in terms of the reliability of the experimental technique, human errors in either conducting the experiments and recording the measured values. In their review, Wang *et al*. reported inconsistencies of experimental values in different databases^9^. Balakin *et al*. also reported the same problem where they found standard deviation (SD) of experimental solubility values of the same compounds as large as 0.5 in LogS units^7^. These errors may result from experimental noise or unintentional misprints. Data verification is important in order to increase the reliability of datasets^9^.

The aim of this study is to curate a large experimental aqueous solubility data, AqSolDB, for data-driven model development. For this purpose, we searched for and collected nine open source datasets on aqueous solubility. In order to merge the datasets, we followed systematic steps of identifier generation by converting CAS numbers and SLN identifiers into SMILES representations, and validation^10–12^. All identifiers were converted to SMILES format and experimental solubility values were all standardized to the LogS units. After we standardized the datasets, we merged all the datasets into one and further grouped them based on their reliability label and the number of occurrences in the merged dataset.

In this *data descriptor*, we provided a general algorithm for selection of the statistically most reliable values from a set of competing values. AqSolDB consists of aqueous solubility values of 9,982 unique compounds, along with some relevant topological and physico-chemical 2D descriptors. Additionally, the dataset contains validated representations of each of the compounds.

AqSolDB is an openly accessible, easy-to-use, and well-structured database of compound. We expect it to serve a broad community as a reference aqueous solubility dataset for the bench-marking of new experimental and physics-based modelling results, and additionally as machine-readable ancillary resource to improve the prediction capability of future machine learning approaches.

## Methods

**Fig. 1:**
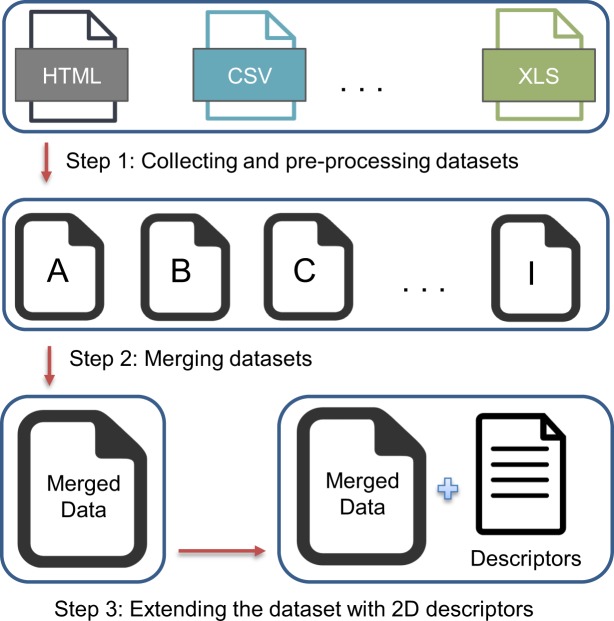
Process diagram of curating solubility dataset.

To curate our dataset we followed three steps. First, we collected nine publicly available aqueous solubility dataset and converted them into a standardized format. Second, we combined datasets into one single dataset by applying a data verification algorithm that selects statistically most reliable experimental value among multiple occurrences. Finally, we added topological and physico-chemical 2D descriptors to the merged dataset. Figure 1 shows the flow of the curation process.

### Step 1: Collecting and pre-processing datasets

**Fig. 2:**
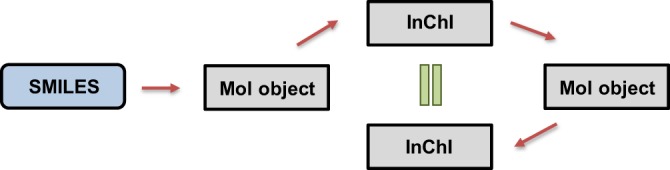
Validation steps of compound representations. Blue box represents the SMILES values from the dataset and gray boxes represent the generated values using RDKit. Red arrows represent the conversion steps and green equal sign represents the validation of consistency.

Solubility data was first collected from nine publicly available datasets as shown in Table 1. A set of three pre-processing steps were applied to each of the datasets in order to standardize the representation format and solubility values in the same units. These steps also describe our exclusion criteria on the basis of unique identifier validation. The steps are as follows:

1. Identifier generation: We chose the SMILES representation as the standard identifier for compounds for our curated dataset. In external datasets, where SMILES representations were not available, we used the name and the CAS Registry Number of compounds as inputs to retrieve the SMILES strings from the Chemical Identifier Resolver web service of the National Cancer Institute (https://cactus.nci.nih.gov/chemical/structure). Lastly, SLN identifiers available from some datasets were converted to SMILES using RDKit open-source cheminformatics software.
2. Unit Conversion: The chosen unit of solubility in this dataset is LogS, where S is the aqueous solubility in mol/L (or M). Units such as g/L and mg/L were converted to LogS using the molecular mass of the compounds.
3. SMILES Validation: In order to ensure consistency and robustness of the SMILES representations, we used InChI representations in the scheme shown in Fig. 2^13^. First, SMILES strings were converted to into RDKit mol objects. If an error occurred during the conversion, the input SMILES string was considered to be invalid. Next, the obtained RDKit mol objects were converted to InChI representations. The InChI representations were used to regenerate the RDKit mol objects. Finally, the thus obtained mol objects were converted back to InChI representations. The original and regenerated InChI were checked for consistency to ensure that the generated InChI were reproducible. This step also validated that both SMILES and InChI representations led to the same RDKit mol object, and thus the chemical compound.

**Table 1:** List of datasets used to curate AqSolDB.

Dataset ID	Original Size	Filtered Size	Compound Representations	Solubility Units
A^14^	14,180	6,110	name, CAS	g/L, mg/L, *μ*g/L
B^15^	5,764	4,651	name, CAS	LogS
C^16^	2,603	2,603	name, SMILES	LogS
D^17^	2,267	2,115	name, CAS	LogS
E^1^	1,291	1,291	name, SMILES, CAS	LogS
F^8^	1,210	1,210	SLN	LogS
G^2^	1,144	1,144	name, SMILES	LogS
H^8^	578	578	SLN	LogS
I^20^	105	94	name, SMILES, InChI	*μ*M

Dataset ID: identifier of the dataset during the curation process. Original Size: number of instances of the dataset when we collected. Filtered Size: number of instances after the pre-process. Compound Representation: available compound representations of the dataset when we collected. Solubility Units: units of experimental solubility values of the dataset.

Table 1 shows the type of information contained in the datasets. Every dataset was processed separately in order to standardize them. The extraction process and standardization methods applied for each dataset, along with the temperature based exclusion criteria, are explained below. We named the datasets from *A* to *I* according to the number of instances they have in descending order.

#### Dataset A (6,110 instances)

Dataset A was obtained from eChemPortal^14^, which is an open source chemical property database developed by the Organisation for Economic Co-operation and Development (OECD). Solubility data was extracted after applying the filters “experimental studies” and “water solubility”. This yielded several lines of bulk text which were then parsed to obtain CAS number, name, and experimental results on solubility including temperature and pH conditions. A total of 14,180 instances were thus obtained and these were further filtered by temperature for a range between 25 ± 5 °C. After filtering, 8,419 instances were obtained. In the identifier generation step, 6,183 of 8,419 compounds were successfully converted into SMILES. Finally, after applying SMILES validation 6,110 instances were obtained.

#### Dataset B (4,651 instances)

Dataset B was downloaded from EPI Suite Data website^15^. This open-source dataset consisted of 5,764 liquid and crystalline organic compounds with the following properties: CAS number, name, molecular weight, water solubility, temperature. SMILES identifiers were successfully generated for 5,367 of these compounds. After that, we filtered the data by temperature between 25 ± 5 °C to obtain 5,206 compounds. In the final step, the InChI and InChIKey were validated to obtain 4,651 compounds.

#### Dataset C (2,603 instances)

Dataset C was collected from the work of Raevsky *et al*.^16^ and it contains solubility data measured at 25 ± 5 °C. The dataset consists of solubility of 2,603 crystalline solid compounds along with SMILES strings. All compounds were successfully recreated after pre-processing steps.

#### Dataset D (2,115 instances)

Dataset D was downloaded from EPI Suite Data website^17^. This open-source dataset consisted of 2,267 liquid and crystalline organic compounds, out of which 2,115 compounds remained after applying the pre-processing steps.

#### Dataset E (1,291 instances)

Dataset E was taken from the work of Huuskonen *et al*.^1^. In this study, the experimental aqueous solubility value measured between 20–25 °C were obtained from the AQUASOL database of the University of Arizona and SCR’s PHYSPROP Database. The extended version of this dataset with 1,291 solubility values and SMILES was downloaded from the Cheminformatics (http://cheminformatics.org/). All compounds were successfully recreated after pre-processing steps.

#### Dataset F (1,210 instances)

Dataset F was taken from the work of Wang *et al*.^8^. They extracted 1,210 compounds from the Beilstein database and sanitized it. However, the dataset contains compound identifiers in only the SLN format^12^. We converted SLN to SMILES representation using RDKit SLN parser. During the conversion 93 of 1,210 compounds could not be produced. Using Molview (http://molview.org/) web tool, we obtained valid SMILES for 93 missing compounds. Name information was collected from NCI Chemical Identifier Resolver service, Molview and SpyderChem^18^ websites. InChI and InChIKey values are produced and validated using the pre-processing steps and all compounds were successfully recreated.

#### Dataset G (1,144 instances)

Dataset G was taken from the work of Delaney *et al*.^2^. The dataset consists of 1,144 small compounds with experimental solubility measured at 25 °C and SMILES information. All compounds were successfully recreated after applying the pre-processing steps.

#### Dataset H (578 instances)

Dataset H was taken from the work of Wang *et al*.^8^, who sanitized the dataset used by Jain and Yalkowsky by removing duplicate entries^19^. This dataset consists of 322 liquid and 256 solid compounds. The dataset contained only SLN as the compound identifiers and after applying the pre-processing steps all compounds are successfully recreated.

#### Dataset I (94 instances)

Dataset I was taken from the Goodman Group website (http://www-jmg.ch.cam.ac.uk/data/solubility/) as the corrected version of solubility challenge^20^. The dataset consists of 105 drug-like compounds with name, SMILES, and solubility information. The solubility values were measured at 25 °C. 11 of 105 crystalline data had to be removed because their solubility values were missing. All compounds were successfully recreated after applying the pre-processing steps.

### Step 2: Merging datasets

**Fig. 3:**
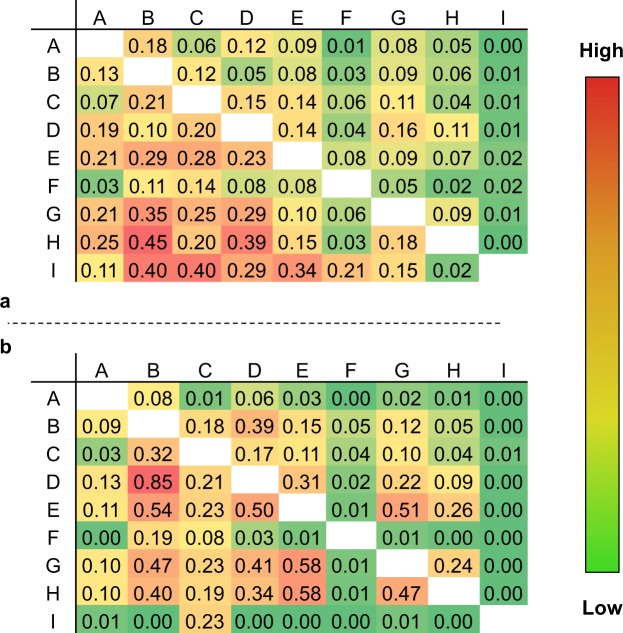
Redundancy matrices showing fractional values for shared compounds between all collected datasets. (**a**) ${M}_{ij}^{d}$ shows fraction of compounds with differing solubility values, and (**b**) ${M}_{ij}^{s}$ shows fraction of compounds with the same solubility values.

The purpose of this step is combining all datasets into the one single repository that contains only unique compounds paired with the most reliable aqueous solubility value. The InChI representation was used to identify compounds uniquely and solubility values within 0.01 LogS units of each other were deemed to be identical. Based on these conditions, a preliminary analysis of the combined repository revealed two different kinds of redundancies - (1) a given compound was found to repeat with a different solubility value, or (2) a given compound was found to repeat with the same solubility value. In order to quantify the relative uniqueness of each of these datasets, redundancy matrices are plotted in Fig. 3, where the rows and columns of these matrices represent the various datasets. Redundant compounds of kind (1) and (2) between any two data sets are represented as fractional values ${M}_{ij}^{d}$ (Fig. 3a) and ${M}_{ij}^{s}$ (Fig. 3b), respectively, where *i* and *j* represent the two datasets in consideration. As an example, the value ${M}_{BA}^{d}$ = 0.13 represents that 13% of the compounds from dataset B can be found in dataset A, but with a different solubility value. In a similar way, the value ${M}_{BA}^{s}$ = 0.09 represents that 9% of the compounds from dataset B can be found in dataset A, but with the same solubility value. The matrix is not symmetric in the fractional representation because of the different sizes of the datasets. While data of kind (2) can be handled simply by removing identical copies, it can be deduced from Fig. 3a about compounds of kind (1) that the datasets possess a high degree of redundancy, which necessitates a strategy for selecting the most reliable value.

**Fig. 4:**
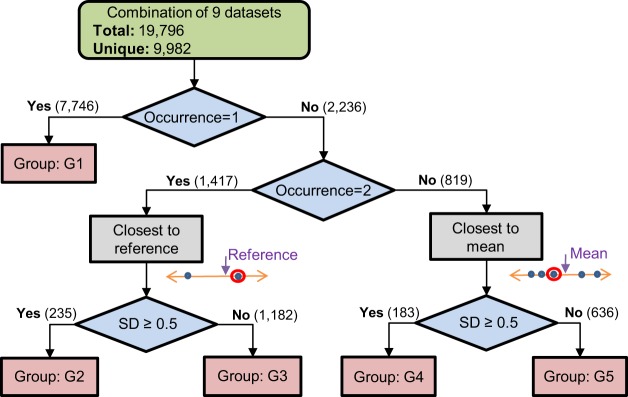
Flowchart of the curation algorithm. Green box represents the initial state. Blue diamond shapes represent a decision according to the number of occurrences of a compound and the SD of multiple occurrences. Pink boxes represent the reliability group. Gray boxes represent the selection method for multiple occurrences. The numbers over the arrows represent the number of unique compound in the corresponding classification path.

There are a total of 19,796 instances in the merged repository with 9,982 unique compounds before redundant values are eliminated. To curate this data set with a unique solubility value for every compound, we design an algorithm to select the most reliable experimental value. The selection is performed by first classifying the compounds into five distinct groups which are defined based on the statistics of occurrence of a compound in the dataset. The flow chart of the curation algorithm is shown in Fig. 4 and described as follows:

1. For every compound in the dataset, the number of occurrences is determined. If the compound has a unique value or multiple values which are within 0.01 LogS units, the value is simply accepted. This step leads to the curation of 7,746 unique instances, which were assigned to group G1.
2. Next, for compounds with occurrence count >1 with different solubility values (819), we used the closest to the mean algorithm to select the value. In this method, the mean value is first calculated then the closest value to mean value among the candidates is selected. If the standard deviation (SD) of the set of values was >=0.5 LogS units, we assigned the compounds to group G4 (183), else to G5 (636).
3. For compounds with exactly 2 values (1,417), the closest to the mean method cannot be applied because mean is always at the middle of the two values. For this case, we used an alternative method, which is closest to the reference. We selected the closest value to an external reference value, which is obtained using the solubility prediction tool ALOGPS^21^. ALOGPS is an open source online solubility prediction tool that is based on artificial neural networks and has an overall error of 0.49 Root Mean Squared Error (RMSE) in LogS units^22^. If the SD of the two values was >=0.5 LogS units, we assigned the compounds to group G2 (235), else to G3 (1,182).

We selected 0.5 as a threshold for degree of agreement between multiple values based on the predictive capabilities of some the state-of-the-art models^3,5,6^. It must also be noted that the average SD of experimental solubility values for a given compound from different sources has been reported to be 0.5 LogS^7,23^. Using this threshold, the grouping of compound into 5 different groups provides a credible way of assessing reliability for data-driven modeling.

### Step 3: Extending the dataset with 2D descriptors

The purpose of this step is extending the information space of compounds by adding basic topological and physico-chemical information. For this purpose, we calculated all the relevant 2D descriptors available from RDKit. The last 17 rows of Table 2 show the name, description and data type of the 2D descriptors.

**Table 2:** List of available information in terms of name, description, and type of each column in the AqSolDB.

Column Name	Description	Type
ID	ID from source (also shows the source)	string
Name	Name of compound	string
InChI	The IUPAC International Chemical Identifier	string
InChIKey	Hashed form of InChI value	string
SMILES	SMILES representation of compound	string
Solubility	Experimental aqueous solubility value (LogS)	float
SD	Standard deviation of multiple occurrences	float
Occurrences	Number of occurrences of compound	integer
Group	Generated reliability group (G1, G2, G3, G4, G5)	string
Mol Wt	Molecular weight	float
Mol LogP	Octanol-water partition coefficient	float
Mol MR	Molar refractivity	float
Heavy Atom Count	Number of non-H atoms	integer
Num H Acceptors	Number of H acceptors	integer
Num H Donors	Number of H donors	integer
Num Heteroatoms	Number of atoms not carbon or hydrogen	integer
Num Rotatable Bonds	Number of rotatable bonds	integer
Num Valence Electrons	Number of valence electrons	integer
Num Aromatic Rings	Number of aromatic rings	integer
Num Saturated Rings	Number of saturated rings	integer
Num Aliphatic Rings	Number of aliphatic rings	integer
Ring Count	Number of total rings	integer
TPSA	Topological polar surface area	float
Labute ASA	Labute’s Approximate Surface Area	float
Balaban J	Balaban’s J index (graph index)	float
Bertz CT	A topological complexity index of compound	float

## Data Records

AqSolDB consists of 9,982 unique compounds. AqSolDB data is stored in the comma-separated values (CSV) format and contains representations, experimental aqueous solubility and calculated 2D descriptor data of all compounds, as described in Table 2. AqSolDB is openly accessible at the Harvard Dataverse Repository^24^.

## Technical Validation

### Analysis of solubility values

**Fig. 5:**
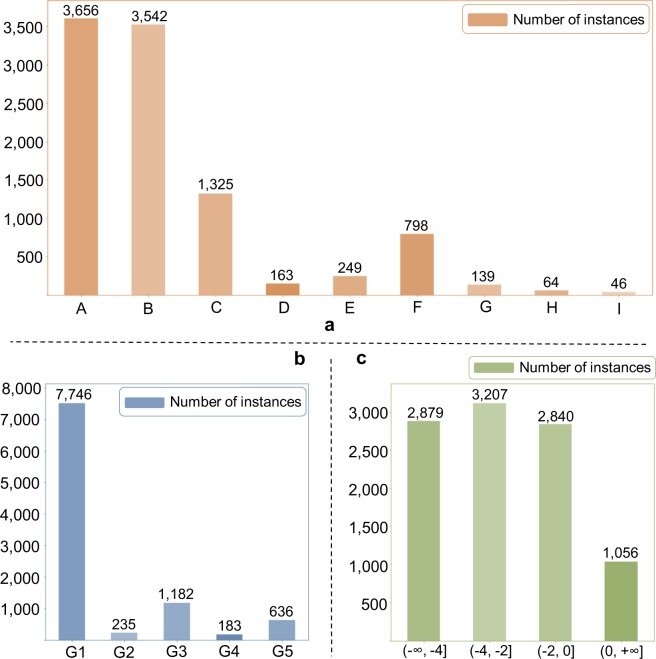
Bar charts for analyzing the curated dataset. (**a**) Distribution of instances according to source dataset. (**b**) Distribution of instances according to reliability group. (**c**) Distribution of instances according to aqueous solubility ranges (LogS).

Compounds can be classified according to solubility values (LogS); Compounds with 0 and higher solubility value are highly soluble, those in the range of 0 to −2 are soluble, those in the range of −2 to −4 are slightly soluble and insoluble if less than −4. Figure 5c shows the distribution of solubility values.

As no information about experimental errors from the original data sources was found to be available, we determined their reliability with a statistical approach. As described in the Methods section, each compound was labeled according to the selection process. Figure 5b shows the distribution of compounds to five groups. The G1 group constitutes the largest part of the data and has been encountered only once in all datasets. These compounds are selected directly and it is not possible to comment on their reliability. G2 and G3 groups are composed of compounds that are found only twice in all datasets. Those with SD values greater than 0.5 were assigned to G2 group and those with small or equal values were assigned to G3 group. G4 and G5 groups are composed of compounds that are found three times or more in all datasets. Using the same process as the previous one, compounds with an SD of greater than 0.5 were included in the G4 group and those with a small or equal value in the G5 group. The difference between the results of the independent experiments shows the reliability of this value. Statistically, due to the fact that when sampling increases, reliability will increase, it can be concluded that G5 group is more reliable than G3 group and G4 group is more reliable than G2 group.

## Usage Notes

It is recommended for users to consider the group description when using the data as input to other models. The availability of the calculated 2D descriptors makes it possible to directly use the data for developing machine learning models. To create a more complex representation of compounds such as graphs or circular fingerprints, we recommend to use RDKit. We provided both SMILES and InChI representations of compounds which are validated and can be easily converted into the RDKit mol object. Further methodological notes on data processing can be found in the Code Ocean repository^25^.

## ISA-Tab metadata file

Download metadata file

## Data Availability

The reproducibility of the curation algorithm can be verified by executing the provided scripts on Code Ocean^25^. The code has been developed and tested using Python 3.5 on Linux operating system and is available under the MIT license. The RDKit cheminformatics software is freely available under the BSD licence (http://www.rdkit.org). ALOGPS 2.1 used for reference value generation is freely available online (http://www.vcclab.org/lab/alogps/).
